# Inclusion of CD80 in HSV Targets the Recombinant Virus to PD-L1 on DCs and Allows Productive Infection and Robust Immune Responses

**DOI:** 10.1371/journal.pone.0087617

**Published:** 2014-01-27

**Authors:** Kevin R. Mott, Sariah J. Allen, Mandana Zandian, Omid Akbari, Pedram Hamrah, Hadi Maazi, Steven L. Wechsler, Arlene H. Sharpe, Gordon J. Freeman, Homayon Ghiasi

**Affiliations:** 1 Center for Neurobiology and Vaccine Development, Ophthalmology Research, Department of Surgery, Cedars-Sinai Burns & Allen Research Institute, Cedars-Sinai Medical Center, Los Angeles, California, United States of America; 2 Department of Molecular Microbiology and Immunology, Keck School of Medicine, University of Southern California, Los Angeles, California, United States of America; 3 Massachusetts Eye & Ear Infirmary, Department of Ophthalmology, Harvard Medical School, Boston, Massachusetts, United States of America; 4 Gavin Herbert Eye Institute, the Department of Ophthalmology, the Department of Microbiology and Molecular Genetics, and the Center for Virus Research, University of California Irvine, School of Medicine, Irvine, California, United States of America; 5 Department of Pathology, Harvard Medical School and Brigham and Women's Hospital, Boston, Massachusetts, United States of America; 6 Department of Medical Oncology, Dana-Farber Cancer Institute, Department of Medicine, Harvard Medical School, Boston, Massachusetts, United States of America; UC Irvine Medical Center, United States of America

## Abstract

CD80 plays a critical role in stimulation of T cells and subsequent control of infection. To investigate the effect of CD80 on HSV-1 infection, we constructed a recombinant HSV-1 virus that expresses two copies of the *CD80* gene in place of the latency associated transcript (LAT). This mutant virus (HSV-CD80) expressed high levels of CD80 and had similar virus replication kinetics as control viruses in rabbit skin cells. In contrast to parental virus, this CD80 expressing recombinant virus replicated efficiently in immature dendritic cells (DCs). Additionally, the susceptibility of immature DCs to HSV-CD80 infection was mediated by CD80 binding to PD-L1 on DCs. This interaction also contributed to a significant increase in T cell activation. Taken together, these results suggest that inclusion of CD80 as a vaccine adjuvant may promote increased vaccine efficacy by enhancing the immune response directly and also indirectly by targeting to DC.

## Introduction

Dendritic cells (DCs) are bone marrow-derived cells that are involved in antigen capture, processing, and presentation and are the most powerful of the antigen presenting cells (APCs), playing a key role in triggering the immune system against infectious agents [1]–[6]. DCs perform crucial roles in linking innate and adaptive immunity and thus play a key role in triggering the immune system against HSV-1 infection [7]–[9].

Recently, we showed that although DCs can be infected by HSV-1, DCs do not support HSV-1 replication and are impervious to cell lysis [10]. However, the mechanism of DCs resistance to HSV-1 replication is not known. In addition, we have reported that in contrast to bone marrow (BM)-derived DCs from wild type mice, DCs isolated from signal transducers and activators of transcription-1 deficient (STAT1^-/-^) mice were susceptible to HSV-1 replication [10]. Binding of CD28 on T cells to CD80 (B7-1) or CD86 (B7-2) on an APC leads to T cell proliferation, differentiation, and cytokine secretion [11]. The CD80 and CD86 molecules are expressed by multiple cell types, including B cells, macrophages, DCs, and T cells [12]–[15].

In addition to CD80 and CD86, the B7 pathways comprise the Programmed Death-1 (PD-1) receptor (CD279) and its two ligands, PD-L1 (B7-H1; CD274) and PD-L2 (B7-DC; CD273) [16], [17]. PD-L1 and PD-L2 expression patterns are different; PD-L1 is constitutively expressed on many cell types such as T cells, B cells, macrophages, DCs, and BM-derived mast cells, while PD-L2 expression is more restricted [18]. Recently we have shown that CD80 binds to PD-L1 and this interaction inhibited T cell proliferation and cytokine production [19].

It was previously shown that DCs were not productively infected despite the fact that DCs express HSV receptors [20]. However, in our hands, few BM-derived DCs expressed HVEM or nectin-1, the two most prominent HSV-1 receptors. The studies presented here utilize a recombinant HSV-1 virus constructed such that it expresses the CD80 gene (HSV-CD80) in an attempt to determine if CD80 expressed by this recombinant virus would bind to PD-L1 expressed on DCs and lead to productive infection and lysis of cells. Our results suggested that viral CD80 binds to PD-L1 on the surface of DCs and facilitates cell infection and lysis. Furthermore, this binding reduced T cell exhaustion *in vitro* independent of CD28. This study lays the framework for a strategy that could be used to prevent and/or significantly reduce T cell exhaustion and thus increase vaccine efficacy against both virus replication in the eye and latency in the TG.

## Results

### Structure of the HSV-CD80 recombinant virus

**Figure 1:**
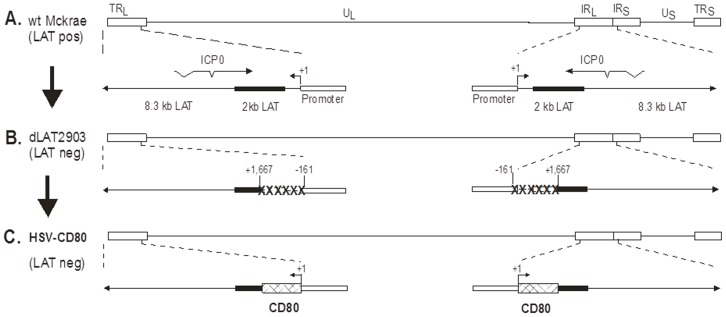
Construction and structure of the HSV-CD80 recombinant virus. (A) The top schematic diagram shows the HSV-1 McKrae genome in the prototypic orientation. TR_L_ and IR_L_ represent the terminal and internal (or inverted) long repeats, respectively, and TR_S_ and IR_S_ represent the terminal and internal (or inverted) short repeats, respectively. U_L_ and U_S_ represent the long and short unique regions, respectively. The solid rectangle represents the very stable 2 kb LAT. (B) dLAT2903 (parental virus for HSV-CD80) has a deletion from LAT nucleotides −161 to +1667 in both copies of LAT and makes no LAT RNA. (C) HSV-CD80 was constructed from dLAT2903 by homologous recombination between dLAT2903 DNA and a plasmid containing the complete LAT promoter and the entire structural CD80 gene (including its 3′ poly(A) signal) as described in Materials and Methods.

Previously we constructed several HSV-1 recombinant viruses expressing various genes using the LAT promoter [21], [22]. In these studies, by using the LAT promoter, we have shown high expression of each gene during both primary and latent infections. This strategy overcame the problems inherent in the temporary expression of various genes provided by immediate early (IE) or HCMV IE promoter. Thus, in this study we constructed a recombinant derivative of HSV-1 strain McKrae that expresses two complete copies of the murine CD80 gene to examine the effects of CD80 expression on HSV-1 infectivity. The genomic structure of the wt HSV-1 strain McKrae is shown schematically in figure 1A. The HSV-1 genome contains a unique long region (U_L_) and a unique short region (U_S_) both of which are flanked by inverted repeats {designated by the open rectangles; terminal and internal repeats long (TR_L_ and IR_L_) and terminal and internal repeats short (TR_S_ and IR_S_)}. The previously described LAT null mutant, dLAT2903 (Fig. 1B), was derived from the HSV-1 McKrae strain [23]. It contains a 1.8 kb deletion in both copies of the LAT gene (one in each of the long repeats). This deletion encompasses 0.2 kb of the LAT promoter and the portion of the LAT gene that encodes the first 1.6 kb of the 8.3 kb primary LAT transcript. The deleted region, designated as “XXXXXX” (Fig. 1B), extends to LAT nt position +1667.

HSV-CD80 was derived from dLAT2903 by insertion of the CD80 gene ORF and restoration of the LAT promoter (Fig. 1C, described in Materials and Methods). The genomic structure of HSV-CD80 was confirmed by restriction enzyme analysis, Southern blot, and partial sequencing (not shown). HSV-CD80 contains the entire sequence of the CD80 gene, including its polyadenylation signal, under the control of the LAT promoter. There is a non-coding region of 47 nt upstream of the first ATG. This is followed by the complete coding region of 948 nt and a 100 nt non-coding region downstream of the CD80 termination codon that contains the CD80 gene polyA signal. Thus, HSV-CD80 and dLAT2903 are both LAT null mutants and HSV-CD80 is identical to dLAT2903 except that the LAT promoter has been restored and it drives expression of the inserted CD80 gene followed by a polyA signal. In the studies below, HSV-CD80 is compared to its parental virus, dLAT2903.

### Replication of HSV-CD80 in RS cells

**Figure 2:**
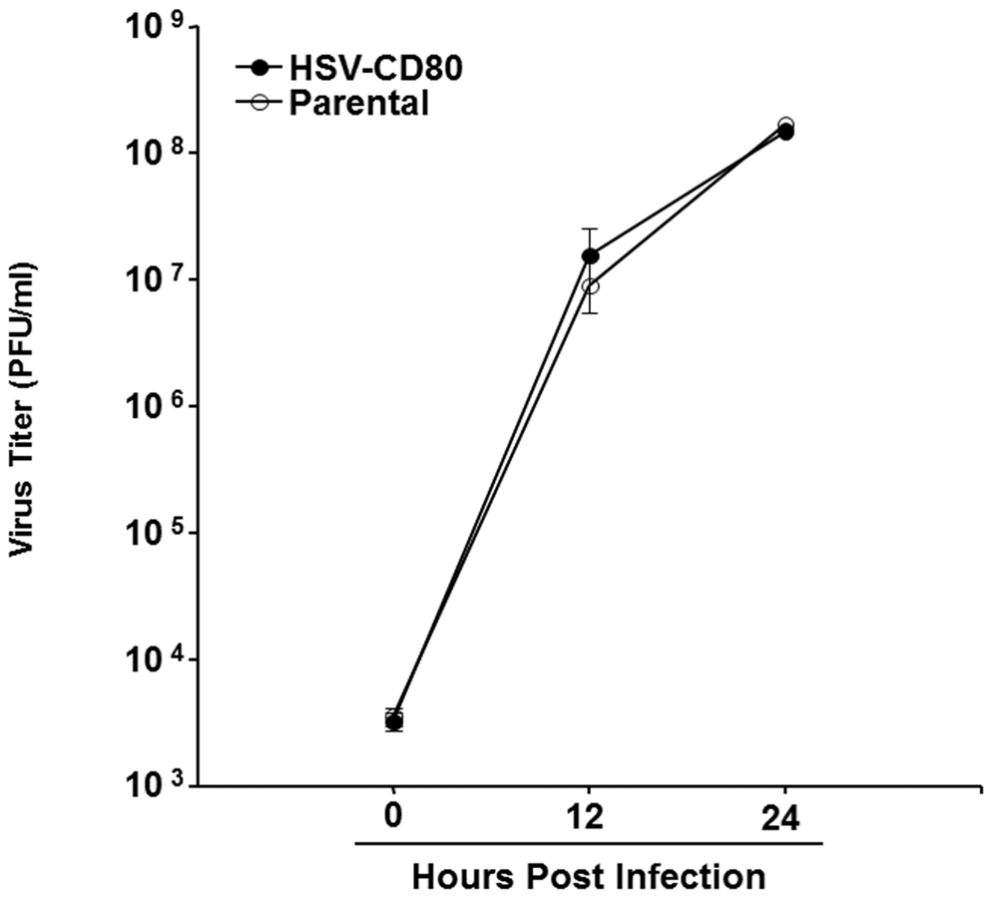
Replication of HSV-CD80 recombinant virus in RS cells. Subconfluent RS cell monolayers in triplicate from two separate experiments were infected with 1/cell of HSV-CD80 or parental dLAT2903 virus as described in Materials and Methods. Total virus was harvested at the indicated times PI by two cycles of freeze-thawing. The amount of virus at each time point was determined by standard plaque assays on RS cells. Each point represents the mean + SEM (n = 6).

RS cells were infected with 1 PFU/cell of HSV-CD80 or parental virus. The monolayers were freeze-thawed at the indicated times, and virus yield was determined as described in Materials and Methods. Replication of HSV-CD80 appeared similar to that of parental virus (Fig. 2; P>0.05 at all-time points). This suggests that the recombinantly expressed CD80 had no direct effect on virus replication in tissue culture. It also strongly suggests that the recombinant HSV-CD80 does not contain any unexpected mutations that effect virus replication in tissue culture.

### HSV-CD80 replication in DCs

**Figure 3:**
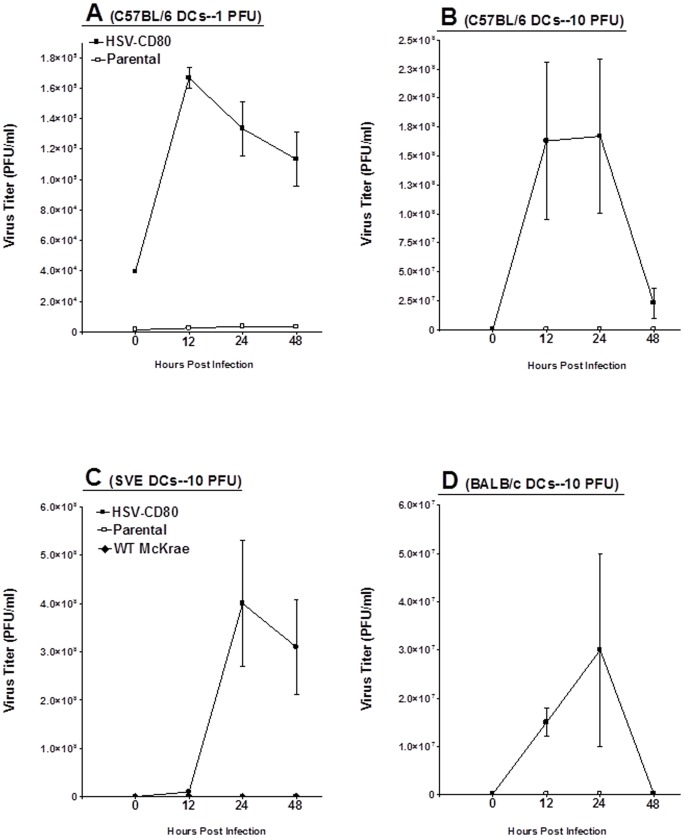
Replication of HSV-CD80 recombinant virus in BM-derived DCs. Subconfluent monolayers of DCs isolated from C57BL/6, 129SVE, and BALB/c mice were infected with 10 PFU/cell of HSV-CD80 or parental virus as described in Materials and Methods. In some experiments DCs from C57BL/6 mice were infected with 1 PFU/cell of each virus, while DCs from 129SVE were infected with 10 PFU/cell of wt HSV-1 strain McKrae. Virus yield was determined at the indicated times PI by standard plaque assays. Panels: A) DCs from C57BL/6 mice were infected at 1 PFU/cell of HSV-CD80 or parental virus; B) DCs from C57BL/6 mice were infected at 10 PFU/cell of HSV-CD80 or parental virus; C) DCs from 129SVE mice were infected at 10 PFU/cell of HSV-CD80, parental, and wt McKrae viruses; and D) DCs from BALB/c mice were infected at 10 PFU/cell of HSV-CD80 or parental virus. Each point represents the mean ± SEM (n = 6) from two separate experiments.

We and others have shown that BM-derived DCs isolated from mice [10] and from blood of humans are refractory to HSV-1 infection [24]–[26]. We tested whether murine BM-derived DCs were similarly resistant to HSV-CD80 infection. DCs isolated from C57BL/6 mice were cultured in the presence of GM-CSF as described in Materials and Methods and then infected with 1 or 10 PFU/cell of HSV-CD80 or parental virus. The kinetics of virus replication were quantitated by determining the amount of infectious virus at various times PI using a plaque assay as described in Materials and Methods. Replication of HSV-CD80 in DCs infected with 1 (Fig. 3A) or 10 (Fig. 3B) PFU was significantly higher than that seen in parental virus infected cells at all-time points. Similar results were obtained when DCs from SVE129 (Fig. 3C) and BALB/c (Fig. 3D) mice were infected with HSV-CD80. Thus, HSV-CD80 infects and replicates with high efficiency in BM-derived DCs isolated from three different strains of mice and the drop of viral titers at later times post infection is most likely related to DC killing by the virus.

### Higher HSV-1 gene expression in HSV-CD80 infected DCs

**Figure 4:**
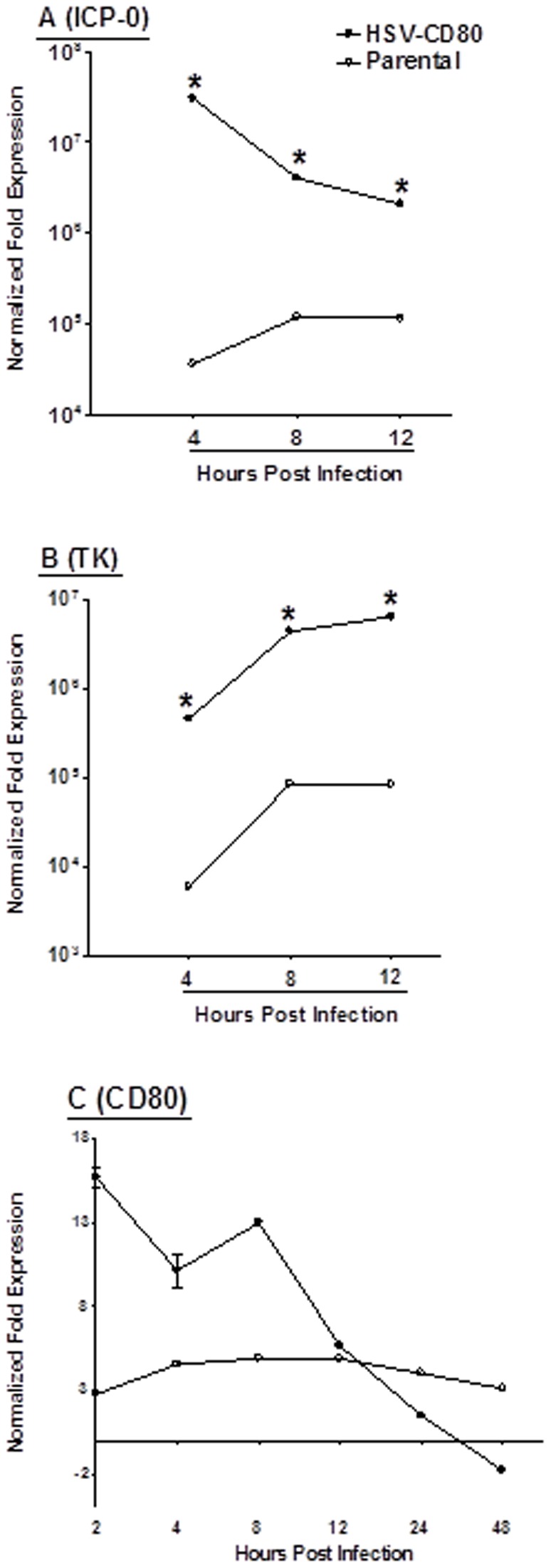
Level of HSV-1 immediate early, early, and CD80 in HSV-CD80 infected DCs. Subconfluent monolayers of DCs from C57BL/6 mice were infected with 1 PFU/cell of HSV-CD80 or parental virus as in Fig. 1. For ICP-0 and TK measurements, cells were isolated 4, 8, and 12 hr PI, while for CD80 measurements cells were harvested 2, 4, 8, 12, 24, and 48 hr PI. Total RNA was isolated and TaqMan RT-PCR was performed using ICP0-, TK-, and CD80-specific primers as described in Materials and Methods. ICP0 and TK mRNA levels were normalized in comparison to each transcript at 0 hr PI, while CD80 mRNA level was normalized to the level of CD80 in mock infected DCs. GAPDH was used as internal control. Each point represents the mean ± SEM (n = 6) from two separate experiments. Panels: A) ICP-0; B) TK, and C) CD80.

To confirm that the above viral replication results are reflected in HSV-1 gene expression, we used ICP0 as an indicator of immediate-early (IE) gene expression and TK as an indicator of early (E) gene expression. We infected DCs with 1 PFU/cell of HSV-CD80 or parental virus and harvested cells at 4, 8, 12 hr PI. TaqMan RT-PCR of isolated total RNA was used to compare ICP0 and TK mRNA levels to baseline levels (just prior to infection). Cellular GAPDH mRNA was used as an internal control. Between 4 hr and 12 hr PI, ICP0 (Fig. 4A, ICP0) and TK (Fig. 4B, TK) transcripts were significantly higher in HSV-CD80 infected DCs than in parental virus infected DCs (p<0.0001 at all three time points). These results were consistent with increased viral replication in HSV-CD80 infected DCs compared to parental virus infected DCs (Fig. 3). The drop in ICP-0 expression at later times post infection in HSV-CD80 infected DCs is probably associated with DCs killing by the virus.

To confirm expression of CD80 by HSV-CD80, the expression of CD80 mRNA was determined. DCs were infected with HSV-CD80 or parental virus at a multiplicity of 1 PFU/cell. Infected cells were collected 2, 4, 8, 12, 24, and 48 hr PI. TaqMan RT-PCR was performed on isolated total RNA to detect CD80 transcripts. As expected, the level of CD80 RNA in HSV-CD80-infected DCs was significantly higher than in parental-infected DCs at 2, 4 and 8 hr PI (Fig. 4C; p<0.001), indicating that the presence of two copies of CD80 in HSV-CD80 leads to increased expression of CD80 during the early period of infection. The reason for the decline in CD80 transcript at 12, 24, and 48 hr PI is not presently understood, but may be due to decreased LAT promoter activity and/or cell lysis of infected cells.

**Figure 5:**
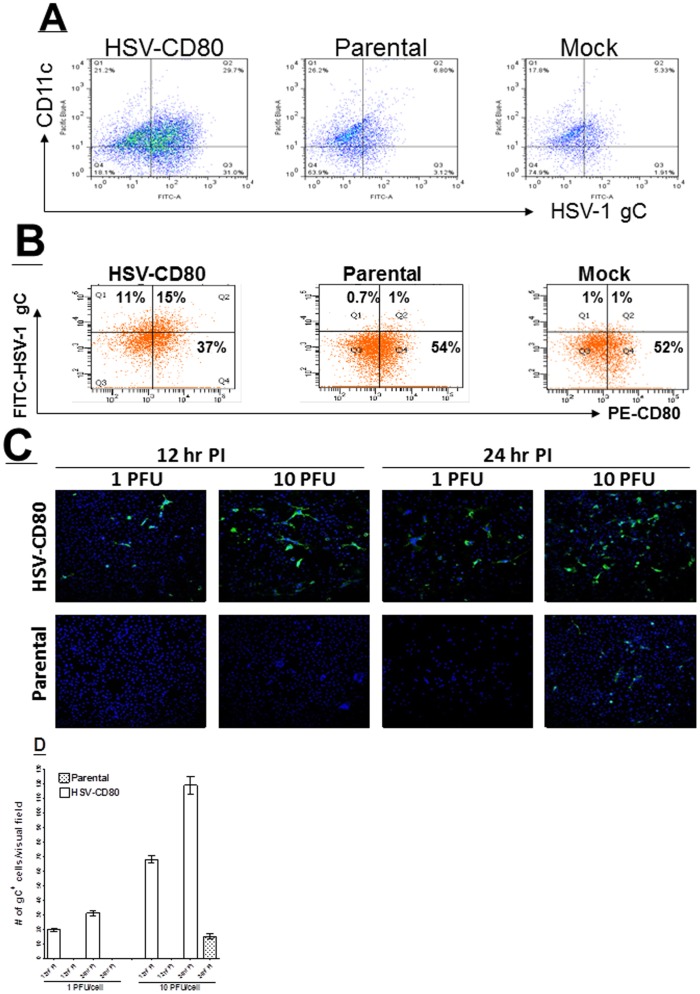
Expression of CD80 by HSV-CD80 in infected DCs. A) Detection of HSV-1 gC in infected DCs. Subconfluent monolayers of DCs isolated from C57BL/6 mice were infected with 1 PFU/cell of HSV-CD80, parental virus, or mock infected. At 24 hr PI, cells were harvested and reacted with anti-CD11c and anti-HSV-1 gC antibodies and FACS analysis was performed (see Materials and Methods). Experiments were repeated twice; B) FACS analyses of infected DCs for expression of HSV-1 gC and CD80. Monolayers of DCs were treated as above. At 24 hr PI, cells were harvested and reacted with anti-CD11c, anti-HSV-1 gC, and anti-CD80 antibodies and FACS analysis was performed. CD11c^+^ cells were gated for expression of HSV-1 gC and CD80. Experiments were repeated twice; C) Immunostaining of HSV-CD80 infected DCs. DCs were infected with 1 or 10 PFU/cell of HSV-CD80 or parental virus. At 12 and 24 hr PI, cells were stained with anti-HSV-1 gC antibody and subjected to IHC as descried in Materials and Methods; and D) Quantification of photomicrographs from C. Different areas of 3 slides/virus/pfu/time point from IHC described above were imaged and the number of HSV-1 gC^+^ cells was counted. Each point represents the mean ± SEM of HSV-1 gC^+^ DCs from 12 images.

Since increased mRNA levels suggest increased protein expression, increased virus protein expression was confirmed by FACS analyses. DCs were infected as above with 1 PFU/cell. At 24 hr PI, the number of HSV-1 infected DCs expressing CD80 was determined using flow cytometry. Approximately 29.7% of DCs infected with HSV-CD80 co-expressed CD11c and HSV-gC compared with 6.8% and 5.3% in parental and mock infected DCs, respectively (Fig. 5A). In addition, 31% of cells were CD11C-negative but gC-positive in the HSV-CD80 group compared with 3.1% and 1.9% in parental and mock infected DCs, respectively (Fig. 5A). In addition, 15% of DCs infected with HSV-CD80 were positive for expression of both CD80 and HSV-1-gC compared with only 1% for the parental or mock groups (Fig 5B).

The RT-PCR and FACS analyses were additionally confirmed by immunostaining. Briefly, DCs were grown on Lab-Tex chamber slides and infected with 1 or 10 PFU/cell of HSV-CD80 or parental virus for 12 or 24 hr. At both 12 and 24 hr PI, gC expression in HSV-CD80 infected DCs was higher with 10 PFU versus 1 PFU (Fig. 5C). In contrast, no gC-positive cells were detected in DCs that were infected with 1 or 10 PFU/cell of parental virus (Fig. 5C). Similarly no gC positive cells were detected in DCs that were infected with 1 PFU of parental virus for 24 hr, while some positive cells were detected at 10 PFU/cell (Fig. 5C, parental, 10 PFU). Quantitative image analyses of HSV-1 gC stained slides described in figure 5C revealed significant increases in HSV-CD80 versus parental virus for both time points and infectious doses (Fig. 5D, p<0.001). Thus, these results provide confirmatory evidence that HSV-CD80 infects DCs more efficiently than parental virus. We also looked at the maturation state of DCs (Figure S1). DCs were prepared as above and stained with anti-CD11c, anti-CD69 and anti-CD83 antibodies. As expected significant numbers of DCs appeared to be immature DCs (Figure S1).

### Colocalization of HSV-CD80 expressed CD80 to PD-L1 is required for HSV-1 infectivity

**Figure 6:**
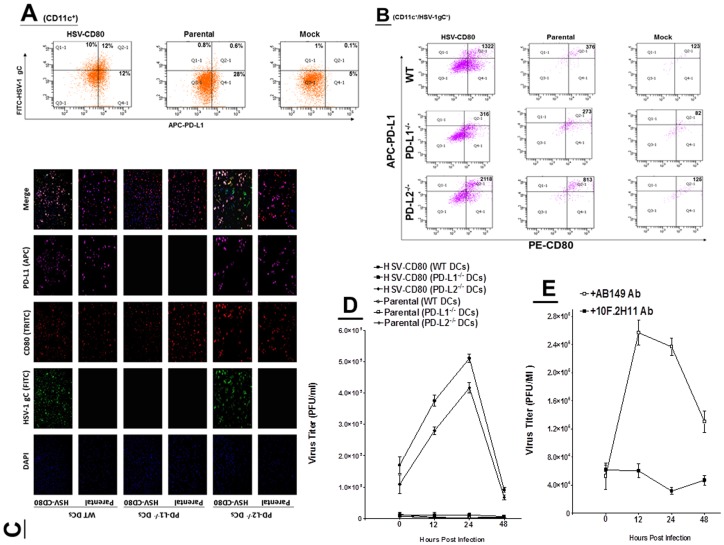
Colocalization of PD-L1 on DCs to CD80 expressed by HSV-CD80 and its effect on virus replication. A) FACS analyses of infected WT DCs. Subconfluent monolayers of DCs isolated from WT C57BL/6 mice were infected with 1 PFU/cell of HSV-CD80, parental virus, or mock infected. At 24 hr PI, cells were harvested and reacted with anti-CD11c, anti-HSV-1 gC, and anti-PD-L1 antibodies and three-color FACS analysis was performed. CD11c^+^ gated cells were analyzed for expression of HSV-1 gC and PD-L1. Experiments were repeated twice; B) FACS analyses of DCs from knockout mice. DCs from C57BL/6 WT, C57BL/6-PD-L1^-/-^ and C57BL/6-PD-L-2^-/-^ mice were infected with 1 PFU/cell of HSV-CD80, parental virus, or mock infected. At 24 hr PI, cells were harvested and reacted with anti-CD11c, anti-CD80, and anti-PD-L1 antibodies and three-color FACS analysis was performed. CD11c^+^ gated cells were analyzed for expression of CD80 and PD-L1. Experiments were repeated twice; C) Immunostaining of DCs from knockout mice. DCs from WT BALB/c, BALB/c-PD-L1^-/-^ and BALB/c-PD-L-2^-/-^ mice were infected with 1 PFU/cell of HSV-CD80 or parental virus. At 24 hr PI, cells were reacted with anti-HSV-1 gC, anti-CD80, and anti-PD-L1 antibodies. DAPI is shown as a nuclear counter-stain; D) Replication of HSV-CD80 in DCs isolated from knockout mice. Subconfluent monolayers of DCs isolated from WT BALB/c, BALB/c-PD-L1^-/-^ and BALB/c-PD-L-2^-/-^ mice were infected with 1 PFU/cell of HSV- CD80 or parental virus as described in Materials and Methods. Virus yield was determined at the indicated times by standard plaque assays. Each point represents the mean ± SEM (n = 6) from two separate experiments; and E) Replication of HSV-CD80 in WT C57BL/6 DCs in presence of blocking antibody. Subconfluent monolayers of DCs were incubated with 10F.2H11 antibody, irrelevant antibody, or no antibody and infected with 1 PFU/cell of HSV-CD80 as described in Materials and Methods. Virus yield was determined at the indicated times by standard plaque assays. Each point represents the mean ± SEM (n = 6).

Previously we have shown that CD80 binds to PD-L1 [19]. To confirm that HSV-CD80 expressed CD80 binds to PD-L1 on DCs, DCs were infected with HSV-CD80, parental virus, or mock-infected as described above. At 24 h PI, the infected DCs were isolated and three-color FACS analyses performed using anti-CD11c, anti-PD-L1, and anti-HSV-1 gC antibodies. DCs were gated on CD11c+ cells and analyzed for expression of PD-L1 and HSV-1 gC (Fig. 6A). The level of co-expression of HSV-1 gC and PD-L1 in HSV-CD80 infected CD11c^+^ cells was higher (12%) than in parental virus infected cells (0.6%) or mock (0.1%) (Fig. 6A).

To determine if the presence of PD-L1 is required for colocalization and thus infectivity of DCs by HSV-CD80, DCs from WT, PD-L1^-/-^, and PD-L2^-/-^ mice were infected with HSV-CD80, parental virus, or mock-infected and analyzed by FACS 24 hr PI as described above. As expected, HSV-CD80 infected DCs from PD-L1^-/-^ mice had few PD-L1^+^/CD80^+^ DCs but both WT and PD-L2^-/-^ had substantial numbers of PD-L1^+^/CD80^+^ DCs (Fig. 6B, HSV-CD80). In DCs infected with parental virus or mock, the number of PD-L1^+^/CD80^+^ DCs was low from WT, PD-L1^-/-^, and PD-L2^-/-^ mice (Fig. 6B).

DCs from WT, PD-L1^-/-^, and PD-L2^-/-^ mice were grown on Lab-Tex chamber slides and infected with 1 PFU of HSV-CD80 or parental virus for 24 hr. Infected DCs were stained with anti-HSV-1 gC, anti-CD80, and anti-PD-L1 antibodies. Only WT and PD-L2^-/-^ DCs infected with HSV-CD80 were positive for HSV-1 gC whereas PD-L1^-/-^ DCs infected with HSV-CD80 were not (Fig. 6C). Consistent with the above results, DCs from WT, PD-L1^-/-^, and PD-L2^-/-^ mice infected with parental virus were negative for expression of HSV-1 gC (Fig. 6C, HSV-1 gC (FITC)). However, DCs from all groups were positive for expression of CD80 (Fig. 6C, CD80 (TRITC)), while DCs from WT and PD-L2^-/-^ mice (but not from PD-L1^-/-^mice) were positive for expression of PD-L1 (Fig. 6C, PD-L1 (APC)). Merging HSV-1 gC, CD80, and PD-L1 show that only DCs from WT and PD-L2^-/-^ and not PD-L1^-/-^ mice infected with HSV-CD80 were gC^+^CD80^+^PD-L1^+^ (Fig. 6C, Merge).

To confirm the above results (Fig. 6A–C) that the presence of PD-L1 is required for infection of DCs by HSV-CD80, DCs from WT, PD-L1^-/-^, and PD-L2^-/-^ mice were isolated and infected with 1 PFU/cell of HSV-CD80 or parental virus. The kinetics of virus replication were quantitated by determining the amount of infectious virus at 0, 12, 24, and 48 PI using a plaque assay as described in Materials and Methods. Replication of WT and PD-L2^-/-^ DCs infected with HSV-CD80 was significantly higher than that seen in PD-L1^-/-^ or parental virus infected cells at all-time points (Fig. 6D; p<0.0001). Thus, the presence of PD-L1 is required for DCs to be efficiently infected with HSV-CD80.

In figure 6D we have shown that DCs from PD-L1^-/-^ mice but not from WT or PD-L2^-/-^ mice are refractory to infection by HSV-CD80 recombinant virus. The lack of effective infection in DCs from PD-L1^-/-^ mice could be due to the absence of some other factors for virus entry, thus to rule out these possibilities, DCs from WT mice were incubated with anti-PD-L1 mAb, 10F.2H11, or irrelevant antibody. This mAb specifically blocks interaction of PD-L1 with CD80 but not with PD-1 [19]. Virus replications were quantitated at 0, 12, 24, and 48 PI using a standard plaque assay. Replication of DCs incubated with irrelevant antibody was the same as control with no antibody and both were significantly higher than that seen in DCs incubated with 10F.2H11 mAb at all-time points (Fig. 6E; p<0.0001). Thus, colocalization of CD80 to PD-L1 is required for DCs to be efficiently infected with HSV-CD80.

### Reduction of T cell exhaustion in the presence of DCs infected with HSV-CD80

**Figure 7:**
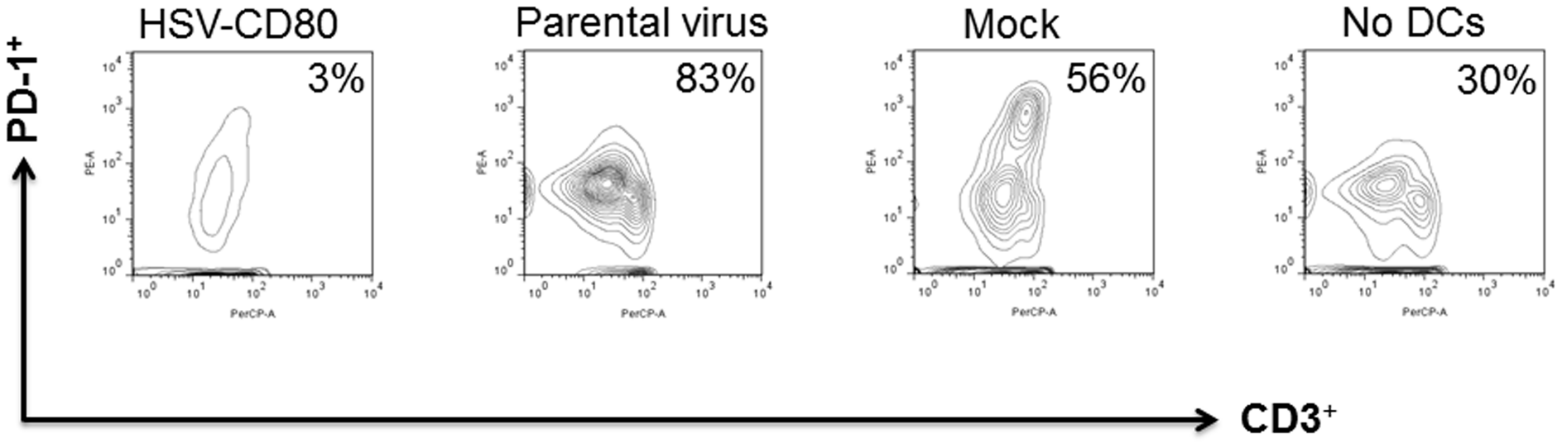
Coculture of HSV-CD80 infected DCs with naive WT T cells reduces CD3^+^PD-1^+^ expression. DCs from WT C57BL/6 mice were infected with 1 PFU/cell of HSV-CD80, parental virus, or mock-infected. At 24 hr PI, the infected DCs were incubated with T cells isolated from naive WT C57BL/6 mice at a 1:1 ratio. As a control some T cells were incubated without DCs. FACS analyses were carried out 24 post incubation using anti-CD3 and anti-PD-1 antibodies. Graphs show the contour of CD3^+^PD-1^+^ staining for each group. Panels represent HSV-CD80, Parental virus, Mock, and no DCs T cells. Number indicates the percent of PD-1^+^CD3^+^ expressing T cells per treatment. Experiments were repeated twice.

Previously we showed that DC depletion reduces the establishment of latent infection in the TG of ocularly infected mice [8], [9], and this reduction in the level of latency correlates with lower expression of PD-1 [27], [28]. To investigate the potential effect of DCs on T cell exhaustion, we infected BM-derived DCs from wt mice with HSV-CD80, parental virus or mock infected as described above. At 24 hr post incubation, the level of PD-1 expression on T cells was only 3% in HSV-CD80 infected DCs compared with 56% in mock-infected DCs and 83% in parental virus infected DCs (Fig. 7). Approximately 30% of T cells incubated alone (Fig. 7, no DCs) or infected with each virus were positive for expression of PD-1 (not shown). At 48 hr PI the level of T cells expressing PD-1 was 91% in the mock-infected group, 87% in the parental virus infected group, and 46% in the HSV-CD80 virus infected group (not shown). These results suggest that in this assay DCs contributed to an increase of PD-1 expression on T cells and PD-1 expression was reduced by CD80 expression in HSV-CD80 infected DCs.

To determine the possible role of CD28 in the increase of PD-1 expression, DCs isolated from WT mice described above were incubated with T cells isolated from CD28^-/-^ mice at a 1∶1 ratio. The level of PD-1 expression did not change in HSV-CD80 infected DCs in the presence or absence of CD28 (Figure S2). Thus, our results suggest that in the presence of exogenous expression of CD80, CD28 did not alter expression of PD-1 on T cells.

### Increase IFN-γ expression in the presence of DCs infected with HSV-CD80

**Figure 8:**
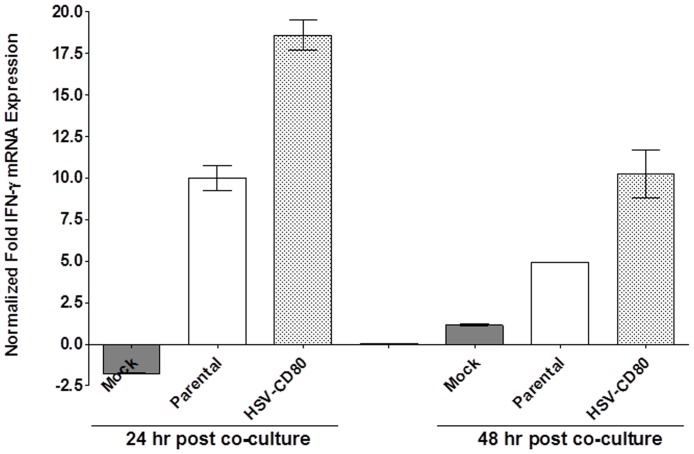
Co-culture of HSV-CD80 infected DCs with naive WT T cells increases IFN-γ expression. DCs from WT C57BL/6 mice were infected with 1 PFU/cell of HSV-CD80, parental virus, or mock-infected as in figure 7. At 24 and 48 hr PI, the infected DCs were incubated with T cells isolated from naive WT C57BL/6 mice at a 1:1 ratio. As a control some T cells were incubated without DCs. At 24 and 48 hr PI, T cells were isolated and quantitative RT-PCR for IFN-γ expression was performed using total RNA. IFN-γ expression in control T cells that were not incubated with DCs was used to estimate the relative expression of IFN-γ transcript in T cells that were co-cultured with mock-infected or infected DCs. GAPDH expression was used to normalize the relative expression of IFN-γ transcript in co-cultured T cells. Each point represents the mean ± SEM from 3 experiments.

The above results suggest that in the presence of exogenous expression of CD80, PD-1 expression on T cells significantly declined. To investigate the effect of CD80 on the level of activation of T cells, T cells were co-cultured for 24 and 48 hr with DCs as above and the relative level of IFN-γ was determined by RT-PCR of total T cells isolated from the co-culture. The results are presented as “fold” increase (or decrease) compared to the baseline mRNA levels in T cells that were not co-cultured with DCs (Fig. 8). At 24 hr post co-culture, the level of IFN-γ expression in T cells incubated with mock uninfected DCs was significantly lower than the levels of IFN-γ expression in T cells incubated with parental or HSV-CD80 infected DCs (Fig. 8; 24 hr post co-culture; p<0.0001). Similarly, the level of IFN-γ expression in T cells incubated with uninfected DCs at 48 hr post co-culture was lower than the levels of IFN-γ expression in T cells incubated with parental or HSV-CD80 infected DCs (Fig. 8; 48 hr post co-culture). At both 24 and 48 hr post co-culture the level of IFN-γ expression was significantly higher in the HSV-CD80 group compared to the parental group or mock group (P<0.0001). However, by 48 hr post co-culture the level of IFN-γ expression in both parental-infected and HSV-CD80-infected groups but not mock-infected group declined due to higher cell death using trypan blue. Overall, our results suggest that the blocking of PD-L1 by CD80 enhances IFN-γ production by T cells.

## Discussion

Herpes simplex virus (HSV) infections are among the most frequent serious viral infections in the U.S. and are considered to be a major health issue in developed countries [29]–[33]. DCs perform crucial roles in linking innate and adaptive immunity and augmenting the immune response to HSV-1 infection [34]. Previously it was reported that freshly isolated peripheral blood monocytes and lymphocytes are refractory to HSV infection [24]–[26], [35]. Similarly, previously we showed that BM-derived DCs could be moderately infected with HSV-1, but the virus does not replicate efficiently [10]. In contrast, other studies showed that while both mature and immature monocyte-derived DCs are infected by HSV-1, only immature DCs produce infectious virus, but at ten-fold lower levels than most cell lines [20], [36]. In this study we showed that BM-derived CD11c^+^ DCs express low levels of CD83/CD69 (Figure S1). During the activation/maturation state, DCs express both CD83 and CD69 that are not detected under resting conditions [37]–[40]. Thus, in this study we have shown that BM-derived DCs are immature and are susceptible to HSV-CD80 infection but not WT HSV-1.

We also report here that HSV-CD80 infects DCs as efficiently as highly permissive RS cells. This attachment and subsequent infection involves colocalization of CD80 to PD-L1 on DCs. This suggests that the non-permissiveness of immature DCs for HSV-1 may be due to low expression of HSV-1 receptors rather than inefficient virus penetration or a block in virus replication. In this study we detected expression of both nectin-1 (Figure S3A) and herpesvirus entry mediator (HVEM) (Figure S3B) on DCs, but less than 10% of the total DC population expressed these two receptors (Figure S3C). Previously it was reported that both immature and mature monocyte-derived DCs (MDDCs) in humans express the HSV receptors nectin-1 and HVEM as determined by FACS using single staining for each receptor [41]. This discrepancy between our results and previous results may be due to the differences in the source of DCs that we used and/or because we looked at expression of these receptors in CD11c^+^ cells.

Because of the critical role that DCs play in linking innate and adaptive immune responses, there is increasing interest in using signals that are known to activate DCs to stimulate and improve vaccine efficacy. However, due to their possible immunotherapeutic potential, the negative aspects of DCs have received far less attention than their role as “immune saviors”. Vaccinia virus abortively infects both mature and immature DCs and blocks their maturation; hence, T cell activation is impaired [42]. By inhibiting the maturation pathway of DCs and inducing their death, Vaccinia virus can subvert the development of efficient antiviral T cell immunity. Similarly, we have shown that DCs contribute to T cell exhaustion [43]. In this study we showed that T cells incubated with DCs infected with HSV-CD80 had less T cell exhaustion (as measured by PD-1 expression) and higher expression of IFN-γ than T cells incubated with mock-infected or parental-infected DCs. The reduced PD-1 could be due to competition between PD-1 and CD80 for binding to PD-L1. However, binding of HSV-expressed CD80 to CD28 did not have any effect on the expression level of CD3^+^PD-1^+^. Nevertheless, the absence of CD28 had a positive effect on the level of T cell exhaustion in mock infected or parental virus infected DCs. In addition, our results confirm recent findings that blocking PD-L1 enhances IFN-γ production by T cells [44].

The roles that PD-L1 plays in T cell activation are controversial. Some studies have demonstrated inhibitory functions for PD-L1 [45], while others have reported that PD-L1 can stimulate T cell proliferation and cytokine production [46]. The functional significance of PD-1 and PD-L1 but not PD-L2 in HSV-1 latency was demonstrated by the significantly reduced amount of HSV-1 latency in PD-1 and PD-L1 deficient mice and by the observation that T cell exhaustion during latent HSV-1 infection correlates with higher levels of latency and reactivation in mice [28]. Thus, HSV-1 may use T cell exhaustion as a means of self-survival. Although T cells maintain an exhausted phenotype after antigen withdrawal and population reexpansion [47], T cell exhaustion can be reversed by anti-PD-L1 antibody [48]-[51]. In this context the potential for autoimmunity induction by DCs, particularly in response to persistent viral infection has been suggested [52]. In our published studies we have shown that depletion of DCs significantly reduced the amount of latency in TG of infected mice, while it had no effect on eye disease, survival, or virus replication in eyes of ocularly infected mice [8], [9]. Consequently, controlling this negative function of DCs by inclusion of CD80 to compete with PD-L1 for its binding to PD-1 might offer potential prophylactic as well as therapeutic targets for the modulation of immune responses in HSV-1-induced latency and reactivation. Also direct binding of CD80 expressed by HSV-CD80 to CD28 on T cells with regards to T cell proliferation and cytokine production cannot be ruled out despite the fact that the level of T cell exhaustion was not altered in presence or absence of CD28. Similar to this study, previously it was shown that PD-1/PD-L1 blockade restored function to T cells [48]–[50], [53]–[55] and this blockade has been shown to have clinical potentials [56], [57]. In conclusion, this study offers a novel strategy for how CD80 can be used to alter T cell exhaustion and restore a functional T cell population.

## Materials and Methods

### Ethics Statement

All animal procedures were performed in strict accordance with the Association for Research in Vision and Ophthalmology Statement for the Use of Animals in Ophthalmic and Vision Research and the NIH *Guide for the Care and Use of Laboratory Animals* (ISBN 0-309-05377-3). Animal research protocol was approved by the Institutional Animal Care and Use Committee of Cedars-Sinai Medical Center (Protocols #2490 and 2309).

### Viruses, cells, and mice

Triple plaque purified wt McKrae (WT), dLAT2903 (LAT-null), and HSV-CD80 (LAT-null) viruses were used in this study. Since dLAT2903 (LAT-null) is the parental virus for HSV-CD80, throughout the study, dLAT2903 virus is called parental virus. Rabbit skin (RS) cells (used for the preparation of virus stocks, the culturing of mouse tear films, and the determination of growth kinetics) were grown in Eagle's minimal essential media (MEM) supplemented with 5% FCS. Six week old wild-type (wt) C57BL/6, C57BL/6-CD28^-/-^, and BALB/c mice were purchased from Jackson Laboratory (Bar Harbor, Maine), while 129SVE mice were purchased from Taconic Farm. C57BL/6-PD-L1^-/-^, C57BL/6-PD-L2^-/-^, BALB/c-PD-L1^-/-^, and BALB/c-PD-L2^-/-^ mice have been reported previously [58], [59] and were bred in-house. Mice were used as a source of bone marrow (BM) for the generation of cultured murine DCs.

### Construction of the CD80 plasmid

Construction of pLAT that was used to insert the CD80 gene was described previously [23]. A plasmid containing the murine CD80 (B7-1) gene was digested with XbaI/HincII (ATCC clone 63369). This insert contained the complete 316 amino acid coding region of the CD80 gene plus 47 and 100 bp of non-coding sequence in its 5′ and 3′ region, respectively. After the addition of a BgLII linker, the insert was ligated into the BamHI site of pLAT and the resulting plasmid was designated pLAT-CD80. This plasmid contains the 1195 bp CD80 gene bounded by 880 and 2989 bp LAT fragments.

### Construction of HSV-CD80 recombinant virus

HSV-CD80 recombinant virus was generated by homologous recombination as we previously described [22], [60], [61]. Briefly, pLAT-CD80 was co-transfected with infectious dLAT2903 DNA using the calcium phosphate method. Viruses from the co-transfection were plated, and isolated plaques were picked and screened for the CD80 gene insertion using restriction digestion and Southern blot analysis. Selected plaques that contained the CD80 gene were plaque purified eight times and reanalyzed by restriction digestion and Southern blot analysis to verify that the CD80 gene was present in the LAT region. A single plaque was chosen for purification and was designated HSV-CD80. This virus contains the CD80 gene in the normal LAT location in the viral genome and is under control of the LAT promoter. Thus, this virus contains two copies of the CD80 gene under the LAT promoter (one in each viral long repeat). We also generated a rescued virus by co-transfection and homologous recombination of DNA from the infectious HSV-CD80 with the original pLAT1.6 plasmid, as above. This rescued virus behaved similarly to that of the dLAT2903 and WT HSV-1 strain McKrae suggesting that it is the expression of CD80 by the HSV-CD80, rather than a defect in the recombinant virus, which mediates the differences between HSV-CD80 with that of dLAT2903 and wt McKrae with regards to their effect on DCs. The kinetics of virus replication between the rescued virus was similar to that of dLAT2903 virus, thus in all experiments described here we used the well characterized dLAT2903 virus also we call it parental virus instead of the HSV-CD80 rescued virus that we made.

### Southern analyses

Briefly, viral DNA was digested with BamHI; the restriction fragments were separated in a 0.9% agarose gel, transferred to Zeta paper, rinsed in 2× SSC (1× SSC is 0.15 M NaCl plus 0.015 M sodium citrate) for 5 min, and cross-linked to the membrane by UV light; and DNA-DNA hybridization was performed with ^32^P-labeled CD80 as previously described [22], [62].

### Virus replication in tissue culture

RS cell monolayers at 70–80% confluency were infected with 1 PFU/cell of parental virus or HSV-CD80. Virus was harvested at various times post infection by subjecting the cell monolayers to two freeze-thaw cycles. Virus titers were determined using standard plaque assays on RS cells as described previously [63].

### DCs culture *in vitro*

Bone marrow (BM) for the generation of mouse DCs was isolated by flushing femurs and tibiae with PBS as we described previously [10]. The cells were centrifuged and resuspended in complete medium supplemented with murine GM-CSF (100 ng/ml; Peprotech, NJ) to enhance replication of DCs [64]. The cells were plated in non-tissue culture plastic Petri dishes (1 bone per 10 cm dish) for 5 days at 37°C with CO_2_. After 5 days, the media was removed, the adherent cells were recovered by incubating the cells for 5 min. at 37°C with Versene (Invitrogen, San Diego, CA). Cells were washed, counted, and plated onto tissue-culture dishes for use the following day.

### Virus replication in DCs

Monolayers of BM-derived DCs were infected with 1 or 10 PFU/cell of HSV-1 strain McKrae, parental virus, or HSV-CD80 for 1 hr at 37°C. Virus was then removed and the infected cells were washed 3X and fresh media was added to each well. The monolayers including media were harvested at various times by freezing at −80°C. Virus was harvested by two cycles of freeze-thawing and infectious virus titers were determined by standard plaque assays on RS cells as we previously described [22], [65].

### Antibody blocking study

Previously we have shown that rat anti-mouse PD-L1 (clone 10F.2H11) antibody blocks interaction of PD-L1 with CD80 but not PD-1 [19], [66]. Monolayers of DCs were incubated with 50 ug/ml of 10F.2H11 antibody, irrelevant antibody of the same isotype (eBioscience), or no antibody. Each 1×107 PFU/ml of HSV-CD80 was similarly incubated with each antibody for 1 hr at 37oC. The mixture of virus-antibody was added to the mixture of DCs-antibody at 0.1 or 1 PFU/cell for 1 hr at 37oC. Virus was then removed and the infected cells were washed 3X and fresh media containing the same concentration of each antibody was added to each well. The monolayers including media were harvested at various times by freezing at −80°C. Virus was harvested by two cycles of freeze-thawing and infectious virus titers were determined by standard plaque assay as above.

### Isolation of T cells

Donor WT C57BL/6 mouse spleens were pooled, and single-cell suspensions were prepared as described previously [67]. Total T cells were isolated using magnetic beads as described by the manufacturer (Miltenyi Biotec, San Diego, CA).

### RNA extraction and cDNA preparation *in vitro*

DCs grown in 24-well plates were infected with 1 PFU/cell of HSV-CD80 or parental virus. RNA preparation was done as we previously described [68]. The RNA yield from all samples was determined by spectroscopy (NanoDrop ND-1000, NanoDrop Technologies, Inc., Wilmington, Delaware) and 1000 ng of total RNA was reverse-transcribed using random hexamer primers and Murine Leukemia Virus (MuLV) Reverse Transcriptase (Applied Biosystems, Foster City, CA), in accordance with the manufacturer's recommendations.

### TaqMan Real-Time PCR

The expression levels of ICP0, TK, and CD80 genes, along with the expression of the cellular IFN-γ, and GAPDH (internal control) genes were evaluated using commercially available TaqMan Gene Expression Assays as we described previously [22], [68]. Primer-probe sets consisted of two unlabeled PCR primers and the FAM™ dye-labeled TaqMan MGB probe formulated into a single mixture. The HSV-1 ICP0 and TK primers and probe used were as follows: 1) ICP0: forward primer, 5′- CGGACACGGAACTGTTCGA-3′; reverse primer, 5′-CGCCCCCGCAACTG-3′; and probe, 5′-FAM-CCCCATCCACGCCCTG-3′ – Amplicon length = 111 bp; and 2) TK: forward primer, 5′- CAGTAGCGTGGGCATTTTCTG-3′; reverse primer, 5′-CCTCGCCGGCAACAAAA-3′; and probe, 5′-FAM-CTCCAGGCGGACTTC-3′ – Amplicon length = 59 bp. For the CD80 (Assay ID MM00711660_m1 - Amplicon length = 117 bp), IFN-γ (ABI assay I.D. Mm00801778_m1 – Amplicon length = 101 bp); and GAPDH internal control (Assay ID m999999.15_G1 - Amplicon length = 107 bp) primers sets from Applied Biosystems were used.

Quantitative real-time PCR was performed as we described previously [68] in triplicate for each sample at each time point. Relative gene expression levels were normalized to the expression of the GAPDH housekeeping gene (endogenous control).

### Flow Cytometric Analysis

BM-derived DCs were infected with 1 PFU/cell of HSV-CD80 and parental virus or mock infected for 24 hr. Infected or mock-infected cells were harvested and stained with anti-CD11c-PacBlue, anti-CD80-PE, anti-PD-L1-APC, anti-HSV-1 gC-FITC, anti-CD83-APC, and anti-CD69-PE from BD PharMingen (San Diego, CA) and Biolegend (San Diego, CA) and then analyzed by FACS as previously described [69].

### Co-culture of infected DCs with T cells

DCs from WT mice were infected with 1 PFU/cell of HSV-CD80, parental virus or mock infected. At 24 hr PI, 1×10^6^ infected or mock-infected DCs were co-cultured with 1×10^6^ T cells from naive WT or CD28^-/-^ mice for 24 and 48 hr. The monolayers were harvested and stained with anti-CD3-PercP-A and anti-PD-1-PE antibodies for FACS analyses (BD PharMingen, San Diego, CA).

### Immunofluorescence

RS cell monolayers grown on Lab-Tex chamber slides were infected with 1 or 10 PFU/cell of parental virus or recombinant HSV-CD80 for 12 and 24 hr. Slides were then blocked for 30 min at room temperature (RT) in PBS-T containing 1% w/v BSA (PBS-TB). Slides were washed, incubated for 1 hr at RT with anti-HSV-1 gC-FITC (Genway, #20-902-170310) antibody. In some experiments DCs isolated from WT, PD-L1^-/-^, and PD-L2^-/-^ mice grown on Lab-Tex chamber slides were infected with 1 PFU/cell of HSV-CD80 or parental virus for 24 hr. Cells were fixed by incubating slides in methanol for 10 min followed by acetone for 5 min at -20×C. Afterwards, slides were rinsed 3× for 5 min each at RT in PBS containing 0.05% v/v Tween-20 (PBS-T). Slides were then blocked for 30 min at RT in PBS-T containing 1% w/v BSA (PBS-TB). Slides were washed, incubated for 1 hr at RT with anti-HSV-1 gC-FITC, anti-CD80, anti-PD-L1, anti-HVEM, and anti-nectin-1 antibodies (Genway, eBioscience, San Diego, CA; abcam, Cambridge, MA). Sections were washed 3× with PBS, air dried and mounted with Prolong Gold DAPI mounting medium (Invitrogen). Images were captured at 20× on independent fluorescence channels using a Nikon C1 eclipse inverted confocal microscope.

## Conclusion

Dendritic cells (DCs) play key roles in host defense against HSV-1 infection. Although DCs can be infected by HSV-1, but DCs are resistant to wild type HSV-1 replication. We report here that, in contrast to wild type HSV-1, DCs were susceptible to infection by a recombinant HSV-1 expressing murine CD80 (B7-1) under the LAT promoter. The susceptibility of DCs to HSV-CD80 infection was due to binding of viral expressed CD80 to PD-L1. Binding of CD80 expressed by HSV-CD80 to PD-L1 reduced PD-L1 interaction with PD-1 on T cells leading to lower T cell exhaustion and higher expression of IFN-γ.

## Supporting Information

Figure S1 **CD83 and CD69 expression in BM-derived DCs.** Subconfluent monolayers of DCs isolated from WT C57BL/6 mice were infected with 1 PFU/cell of HSV-CD80, parental virus, or mock-infected. At 24 hr PI, cells were harvested and reacted with anti-CD11c, anti-CD83 and anti-CD69 antibodies and FACS analysis was performed by gating the CD11c^+^ cells for expression of CD83 and CD69. Experiments were repeated twice.
(PDF)

Figure S2 **Co-culture of HSV-CD80 infected DCs with naive CD28^-/-^ T cells.** DCs from WT C57BL/6 mice were infected with 1 PFU/cell of HSV-CD80, parental virus, or mock-infected. At 48 hr PI, the infected DCs were incubated with T cells isolated from naive C57BL/6-CD28^-/-^ mice at a 1∶1 ratio. As a control some T cells were incubated without DCs (not shown). FACS analyses were carried out using anti-CD3 and anti-PD-1 antibodies. Graphs show the PD-1 staining intensity of CD3^+^ gated T cells. Number indicates the percent of PD-1^+^CD3^+^ expressing T cells per treatment. The left peak represent the PD-1 negative T cells, while the right peak (R9) represent PD-1 positive T cells. Experiments were repeated twice.
(PDF)

Figure S3 **Detection of HSV-1 receptors on the surface of DCs.** Subconfluent monolayers of DCs isolated from WT C57BL/6 mice were grown on Lab-Tek chamber slides and probed with anti-CD11c/anti-HVEM or anti-CD11c/anti-nectin-1 antibodies. Panels: A and B) Representative Photomicrographs of stained DCs. DAPI is shown as a nuclear counter-stain; and C) Quantification of photomicrographs. Different areas of 3 slides were imaged and the numbers of CD11c^+^, CD11c^+^HVEM^+^, and CD11c^+^nectin-1^+^ cells were counted. Each point represents the mean ± SEM from 24 images.
(PDF)
