# Supplementary figures and images for: Inclusion of CD80 in HSV Targets the Recombinant Virus to PD-L1 on DCs and Allows Productive Infection and Robust Immune Responses
Source: PLoS One. 2014 Jan 27;9(1):e87617. doi: 10.1371/journal.pone.0087617 (PMC3903765; doi:10.1371/journal.pone.0087617)

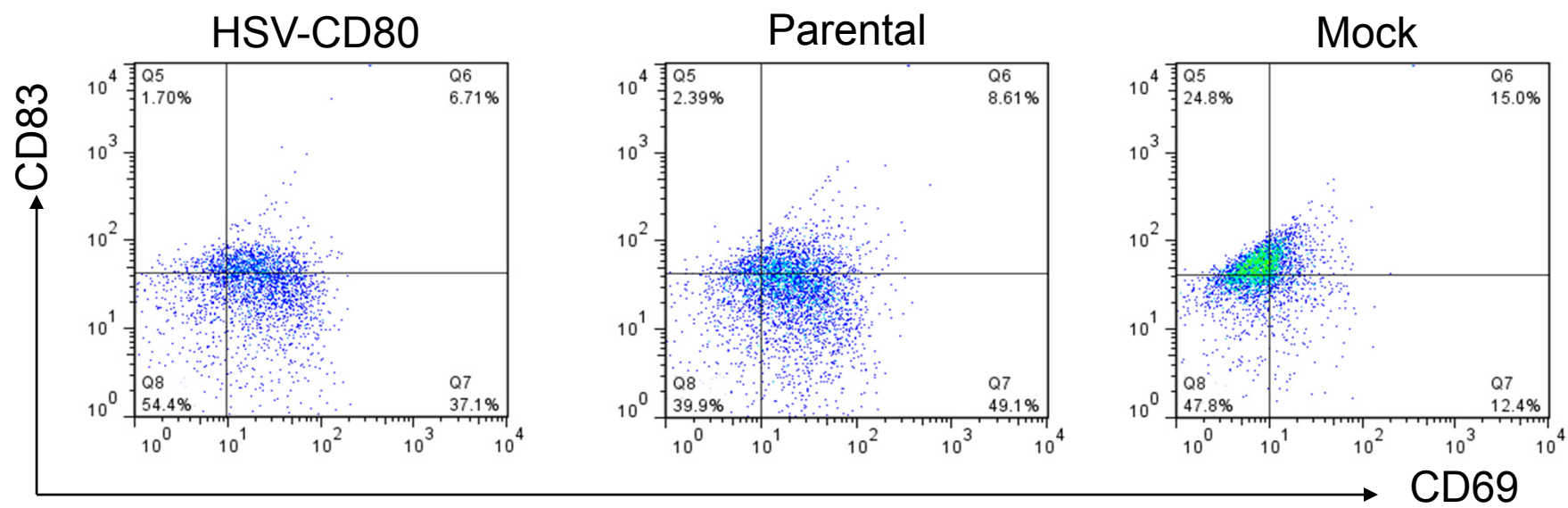

Fig. S1

Supplement: Figure S1 — CD83 and CD69 expression in BM-derived DCs. Subconfluent monolayers of DCs isolated from WT C57BL/6 mice were infected with 1 PFU/cell of HSV-CD80, parental virus, or mock-infected. At 24 hr PI, cells were harvested and reacted with anti-CD11c, anti-CD83 and anti-CD69 antibodies and FACS analysis was performed by gating the CD11c+ cells for expression of CD83 and CD69. Experiments were repeated twice. (PDF) [file pone.0087617.s001.pdf]

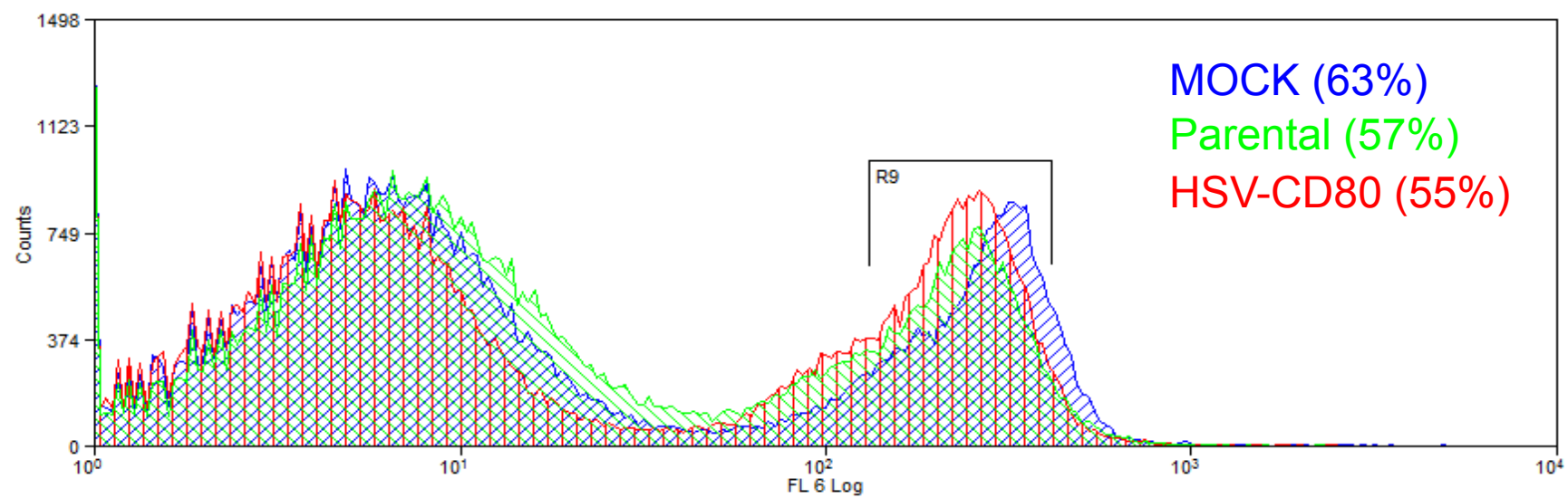

**PD-1<sup>+</sup> gated on CD3<sup>+</sup>**

Fig. S2

Supplement: Figure S2 — Co-culture of HSV-CD80 infected DCs with naive CD28-/- T cells. DCs from WT C57BL/6 mice were infected with 1 PFU/cell of HSV-CD80, parental virus, or mock-infected. At 48 hr PI, the infected DCs were incubated with T cells isolated from naive C57BL/6-CD28-/- mice at a 1∶1 ratio. As a control some T cells were incubated without DCs (not shown). FACS analyses were carried out using anti-CD3 and anti-PD-1 antibodies. Graphs show the PD-1 staining intensity of CD3+ gated T cells. Number indicates the percent of PD-1+CD3+ expressing T cells per treatment. The left peak represent the PD-1 negative T cells, while the right peak (R9) represent PD-1 positive T cells. Experiments were repeated twice. (PDF) [file pone.0087617.s002.pdf]

## A) HVEM

DAPI

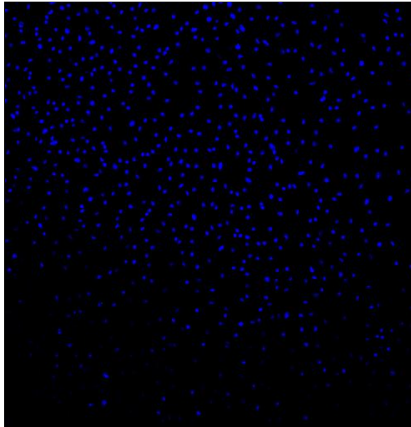

CD11c (FITC)

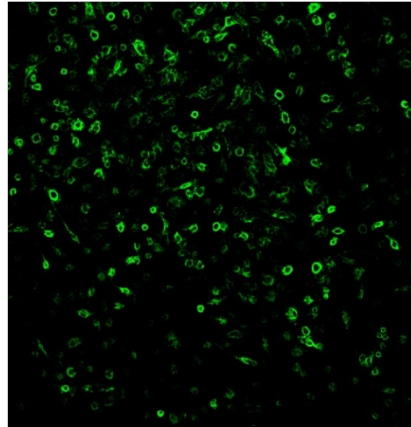

HVEM (TRITC)

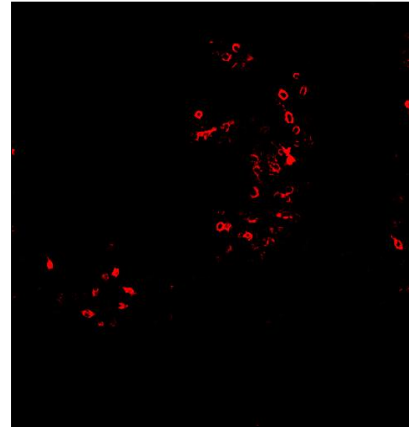

Merge

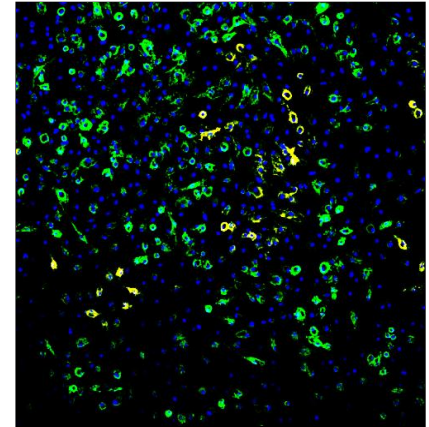

## B) Nectin-1

DAPI

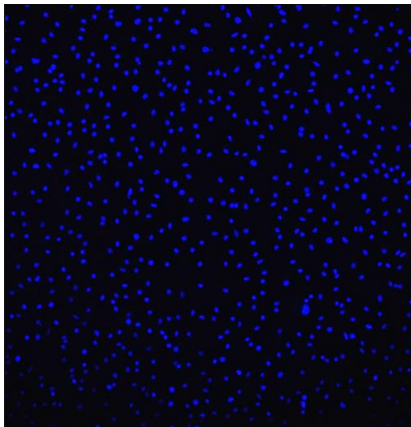

CD11c (FITC)

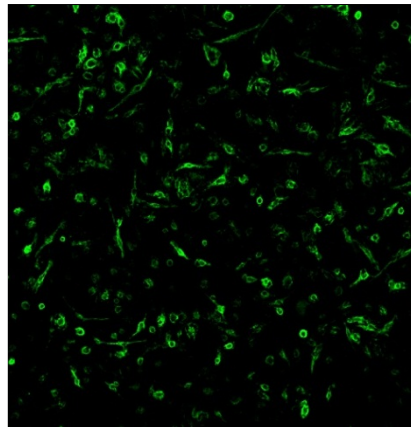

Nectin-1 (TRITC)

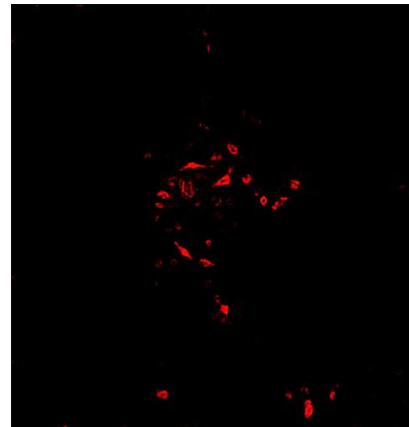

Merge

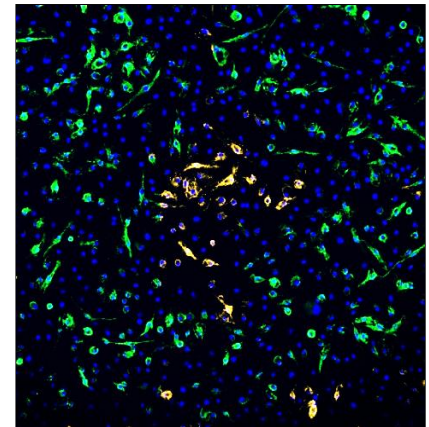

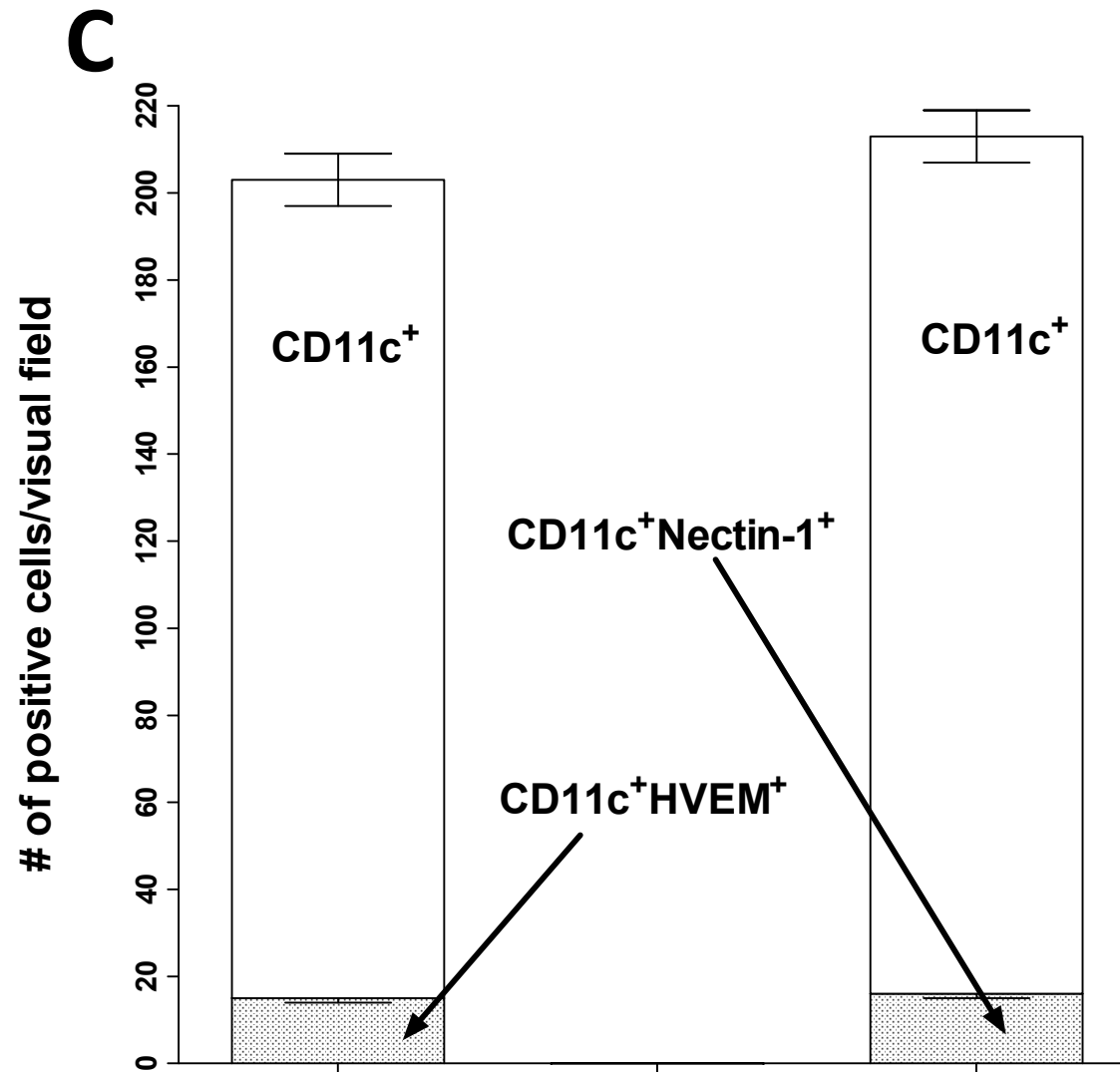

**Fig. S3**

Supplement: Figure S3 — Detection of HSV-1 receptors on the surface of DCs. Subconfluent monolayers of DCs isolated from WT C57BL/6 mice were grown on Lab-Tek chamber slides and probed with anti-CD11c/anti-HVEM or anti-CD11c/anti-nectin-1 antibodies. Panels: A and B) Representative Photomicrographs of stained DCs. DAPI is shown as a nuclear counter-stain; and C) Quantification of photomicrographs. Different areas of 3 slides were imaged and the numbers of CD11c+, CD11c+HVEM+, and CD11c+nectin-1+ cells were counted. Each point represents the mean ± SEM from 24 images. (PDF) [file pone.0087617.s003.pdf]
